# Evaluating a pilot, structured, face-to-face, antimicrobial stewardship, prospective audit-and-feedback program in emergency general surgery service in a community hospital

**DOI:** 10.1017/ash.2023.168

**Published:** 2023-06-05

**Authors:** April J. Chan, Melanie E. Tsang, Bradley J. Langford, Rosane Nisenbaum, Michael Wan, Mark A. Downing

**Affiliations:** 1 Unity Health Toronto, Toronto, Ontario, Canada; 2 University of Toronto, Toronto, Ontario, Canada; 3 Ontario Agency for Health Protection and Promotion, Toronto, Ontario, Canada; 4 Hotel Dieu Shaver Health and Rehabilitation Centre, St. Catharine’s, Ontario, Canada; 5 Li Ka Shing Knowledge Institute, St. Michael’s Hospital, Toronto, Ontario, Canada; 6 Applied Health Research Centre and MAP Center for Urban Solutions, Li Ka Shing Knowledge Institute, St. Michael’s Hospital, Toronto, Ontario, Canada

## Abstract

**Background:**

Prospective audit and feedback (PAF) is an established practice in critical care settings but not in surgical populations. We pilot-tested a structured face-to-face PAF program for our acute-care surgery (ACS) service.

**Methods:**

This was a mixed-methods study. For the quantitative analysis, the structured PAF period was from August 1, 2017, to April 30, 2019. The ad hoc PAF period was from May 1, 2019, to January 31, 2021. Interrupted time-series segmented negative binomial regression analysis was used to evaluate change in antimicrobial usage measured in days of therapy per 1,000 patient days for all systemic and targeted antimicrobials. Secondary outcomes included *C. difficile* infections, length of stay and readmission within 30 days. Each secondary outcome was analyzed using a logistic regression or negative binomial regression model. For the qualitative analyses, all ACS surgeons and trainees from November 23, 2015, to April 30, 2019, were invited to participate in an email-based anonymous survey developed using implementation science principles. Responses were measured using counts.

**Results:**

In total, 776 ACS patients were included in the structured PAF period and 783 patients were included in the in ad hoc PAF period. No significant changes in level or trend for antimicrobial usage were detected for all and targeted antimicrobials. Similarly, no significant differences were detected for secondary outcomes. The survey response rate was 25% (n = 10). Moreover, 50% agreed that PAF provided them with skills to use antimicrobials more judiciously, and 80% agreed that PAF improved the quality of antimicrobial treatment for their patients.

**Conclusion:**

Structured PAF showed clinical outcomes similar to ad hoc PAF. Structured PAF was well received and was perceived as beneficial by surgical staff.

Judicious antimicrobial use is necessary given the increasing rates of antimicrobial resistance worldwide combined with the lack of new antimicrobial agents in the drug-development pipeline.^1^ The decision to use antimicrobials rests primarily on prescribers; therefore, education to bring prescribing behavior in line with current guidelines is essential for improving appropriate antimicrobial prescribing.^2^ However, educational interventions have yielded mixed results. An Australian study showed no change in surgical prophylactic antibiotic prescribing,^3^ and other studies showed reduction in antimicrobial use and improvements in antibiotic choice, timing, and duration.^4–6^ Furthermore, surgeon compliance with antimicrobial stewardship program (ASP) recommendations is poor.^7,8^ Therefore, there is an urgent need for formalized, structured approaches to optimizing antimicrobial therapy in the setting of surgical units.^2^ It has been proposed that the best means to improve antimicrobial stewardship in general surgery should involve collaboration among different specialties, including prescribing surgeons.^2,9^

Prospective audit and feedback (PAF) is an antimicrobial stewardship intervention that aims to maintain prescriber autonomy while increasing the capacity for prescribers to improve the use of antibiotics. Newly introduced PAF programs have been shown to reduce antimicrobial use and to improve patient outcomes.^10^ PAF is more established in critical-care settings and is also used in medical and surgical populations. In one study, PAF in surgical populations led to early detection of inappropriate empirical antibiotic treatment as well as significant reductions in length of stay and duration of antimicrobial therapy.^11^ Another study using PAF with surgeon participation showed a significant decrease in *Pseudomonas aeruginosa* prevalence, coupled with a significance increase in *Klebsiella pneumoniae* prevalence, as well as decreases in meropenem use and resistance.^12^

At our institution, several ASP interventions have been implemented in the surgical population, including prescribing guidelines for common infections and ad hoc PAF for other surgical services. However, compliance with ASP recommendations by surgeons is poor.^8^ Therefore, a more structured form of PAF was pilot tested at our institution, and outcomes were evaluated against ad hoc PAF.

## Methods

The study was conducted at a 400-bed community teaching hospital in Toronto, Canada. An ASP was established at our institution in 2011 with a multimodal approach including PAF, development of guidelines and order sets, microbiology laboratory report optimization, and education. During the study period, staffing consisted of an ASP physician and 2 full-time–equivalent, ASP-trained pharmacists.

Structured PAF consisted of once-weekly, structured, face-to-face PAF with the acute-care surgery (ACS) service. This program was pilot tested from November 23, 2015, to April 30, 2019, at St. Joseph’s Health Centre. The ACS service was chosen for this pilot program because the surgeons and residents do not start their surgeries until the afternoon and would be available for face-to-face PAF. The ACS service is responsible for patients with nonelective general surgery diagnoses (eg, appendicitis, cholecystitis, bowel obstruction, perforated viscus, etc) and was established in 2015 to streamline care for acutely ill patients in need of timely surgical intervention. These structured PAF sessions were attended primarily by the ASP pharmacist and ACS resident with occasional attendance from the ASP physician, the ACS staff physician, and/or the surgical ward pharmacist. These sessions lasted ∼10–15 minutes on Monday mornings. The ASP pharmacist led these discussions by (1) identifying all ACS patients on antimicrobial therapy; (2) assessing indication for treatment and appropriateness of prescribed regimen based on relevant laboratory values, cultures, and imaging; and (3) making recommendations to optimize antimicrobial therapy. Starting April 30, 2019, structured PAF was stopped to assess effectiveness of this intervention. Structured PAF was initiated shortly after establishment of the ACS service; prestructured PAF data were lacking. Starting May 1, 2019, ad hoc PAF was reimplemented. It focused on select ACS patients on targeted antimicrobials highly associated with *Clostridioides difficile* infection and/or broad-spectrum antimicrobials: third-generation cephalosporins, fluoroquinolones, piperacillin-tazobactam, carbapenems, and clindamycin). This PAF occurred once to twice weekly. Recommendations were made to physicians using written methods (e-mail or note in patient’s chart) or verbal methods (in-person or telephone).

In this mixed-methods study design, the qualitative study period was from November 2019 to February 2020 and quantitative study period was from August 1, 2017, to January 31, 2021. We sought to ascertain the surgeons’ perceptions and experiences with structured PAF, to determine barriers and facilitators to structured PAF, and to evaluate the clinical outcomes of structured PAF compared to ad hoc PAF. This study was approved by the Research Ethics Board of Unity Health Toronto.

### Quantitative study

A quasi-experimental design was used. All systemic antimicrobial orders prescribed by the ACS team from August 1, 2017, to January 31, 2021, were included. The structured PAF study period was from August 1, 2017, to April 30, 2019, whereas the ad hoc PAF period was from May 1, 2019, to January 31, 2021. The primary outcome was change in antimicrobial usage measured in days of therapy per 1,000 patient days (DOT/1,000 PD) for all antimicrobials and targeted antimicrobials ordered by the ACS service during the 21 months of structured PAF compared to the 21 months of ad hoc PAF. Targeted antimicrobials included third-generation cephalosporins, fluoroquinolones, piperacillin-tazobactam, carbapenems, and clindamycin. Secondary outcomes were: (1) incidence of nosocomial *Clostridioides difficile* infection, (2) length of stay, and (3) readmission with 30 days during structured PAF period compared to ad hoc PAF period.

In terms of analysis, interrupted time-series, segmented, negative binomial regression^13,14^ was used to evaluate the changes in antimicrobial usage for all antimicrobials and targeted antimicrobials during structured PAF period compared to ad hoc PAF period. The model parametrization^15^ was used and included an offset equal to the natural log of the monthly number of patient days. For the secondary outcomes, a logistic regression model was used to evaluate nosocomial *C. difficile* infection and readmission within 30 days, and a negative binomial regression model used to evaluate length of stay.

### Qualitative survey

All surgical residents, fellows, and staff surgeons who covered the ACS service from November 23, 2015, to April 30, 2019, were invited to participate by email with a link to an anonymous electronic survey (SurveyMonkey). The email provided information on the study, and completing the survey implied consent to participate in the study. The survey was developed by the investigator group and included 3 demographic questions, 3 items rated on a 5-point Likert scale, and 4 open-ended questions. The items explored surgeons’ perceptions of structured face-to-face PAF and how their antimicrobial knowledge and quality of care for their patients have changed as a result. Quantitative data included data on length of surgical training and practice, number of antimicrobial stewardship rounds attended, attendees of rounds, and responses to the survey questions. Qualitative data included participants’ free-text responses to the most and least useful aspect of structured PAF as well as barriers to and facilitators of judicious use of antimicrobials in surgery. We estimated the participant population to be 40, and we aimed for a response rate from at least 10 participants. A purposeful convenience sampling strategy was employed, and all survey respondents were included. In the analysis, counts and percentages were used for quantitative responses. Responses to free-text questions were analyzed and grouped into common categories.

## Results

### Quantitative study

**Figure 1.**
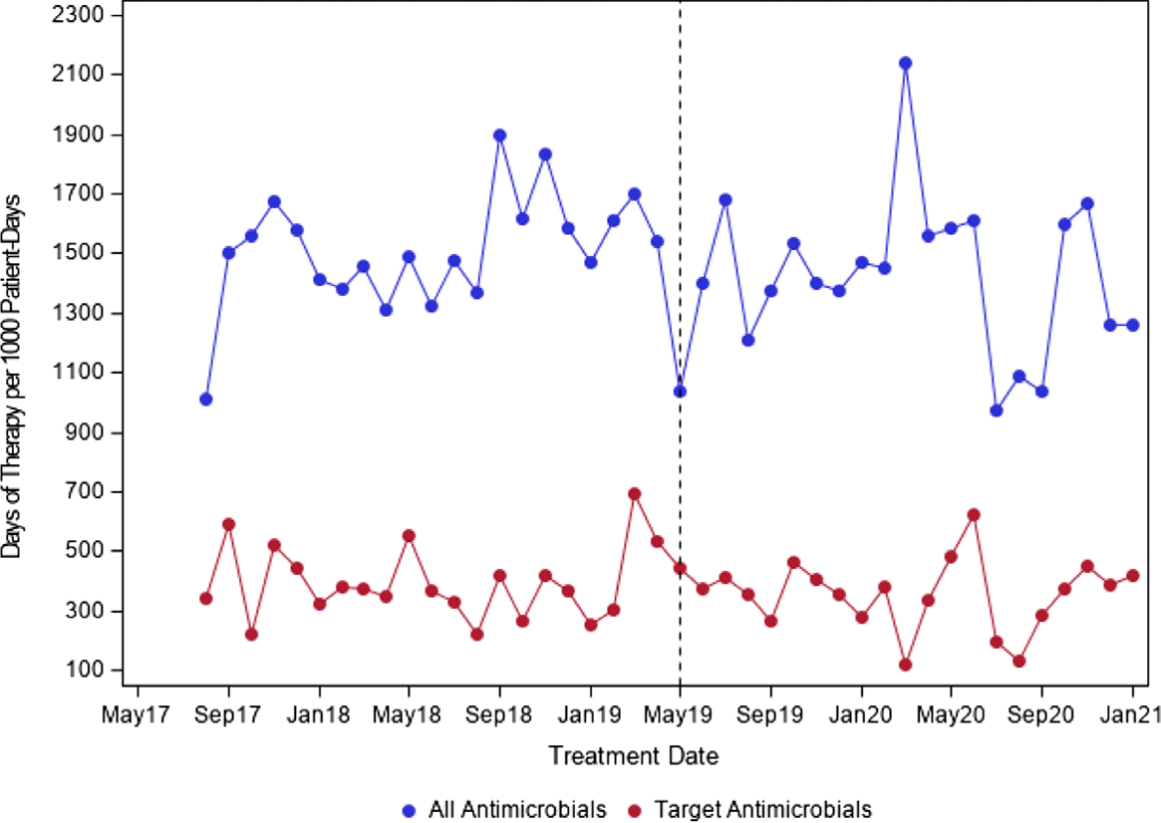
DOT/1000-PD for All and Targeted Antimicrobials.

**Figure 2.**
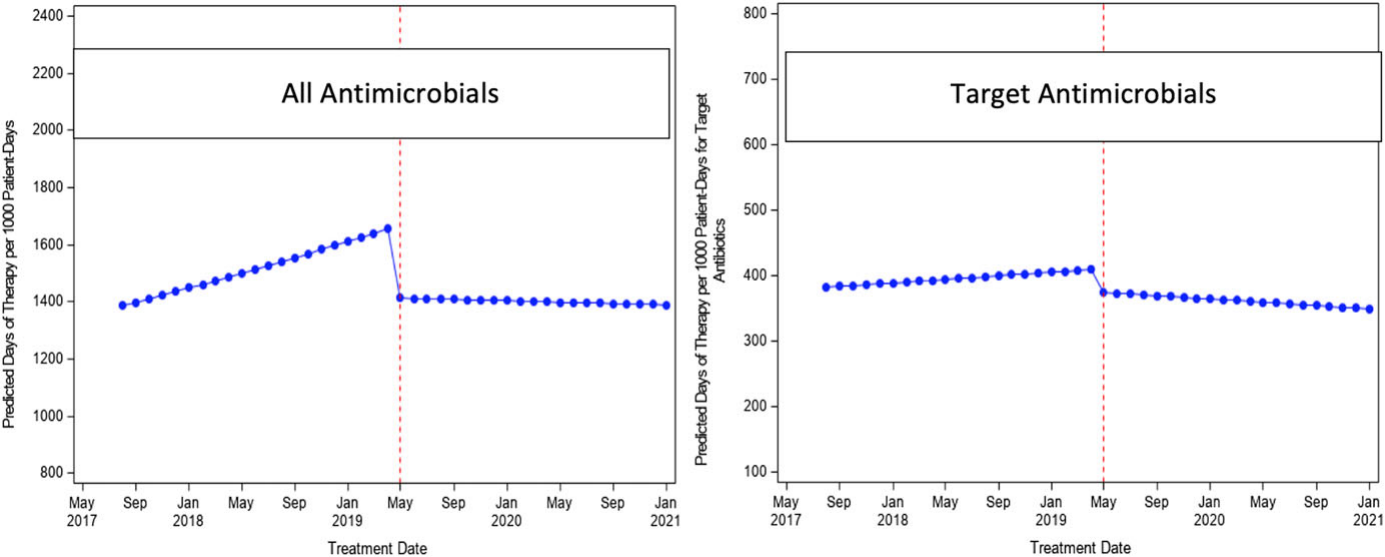
Predicted rates by time using negative binomial models.

We evaluated 776 ACS patients in the structured PAF period and 783 patients in the ad hoc PAF period. For the primary outcome (Table 1), there were no significant changes in level (RR, 0.85; 95% CI, 0.70–1.02) or trend (RR, 0.99; 95% CI, 0.98–1.01) for all antimicrobials. Similarly, for targeted antimicrobials, there was no significant change in level (RR, 0.91; 95% CI, 0.62–1.33) or trend (RR, 0.99; 95% CI, 0.96–1.02). The interrupted time-series, segmented, negative-binomial, regression analysis results are shown in Figures 1 and 2. In terms of secondary outcomes (Table 2), there were no significant differences in readmission with 30 days (OR, 0.87; 95% CI, 0.56–1.35), incidence of *C. difficile* infection (OR, 0.66; 95% CI, 0.06–5.77) or length of stay (RR, 1.07; 95% CI, 0.98–1.17).

**Table 1.** Primary Outcome of Change in Antimicrobial Usage

Outcome	Rate Ratio	95% CI	*P* Value
**All antimicrobials**			
Change in level	0.85	0.70–1.02	0.074
Change in trend	0.99	0.98–1.01	0.209
**Targeted antimicrobials**			
Change in level	0.91	0.62–1.33	0.634
Change in trend	0.99	0.96–1.02	0.664

Note. CI, confidence interval.

**Table 2.** Secondary Outcomes

	Structured PAF N = 776	Ad-hoc PAF N = 783	Odds ratio or Rate ratio (95% CI)	p-value
**Readmission with 30 days (n, %)**				
Yes	45 (5.6)	40 (5.0)	0.87 (0.56-1.35)	0.5457
No	753 (94.4)	766 (95.0)		
***C. difficile infection*** **(n, %)**				
Yes	3 (0.4)	2 (0.3)	0.66 (0.06-5.77)	0.9906
No	795 (99.6)	804 (99.8)		
**Length of stay (days)**				
Mean (standard deviation)	2.7 (3.5)	2.9 (5.0)	1.07 (0.98-1.17)	0.1206
Median (interquartile range)	2.0 (1.0-3.0)	2.0 (1.0-3.0)		

### Qualitative survey

**Figure 3.**
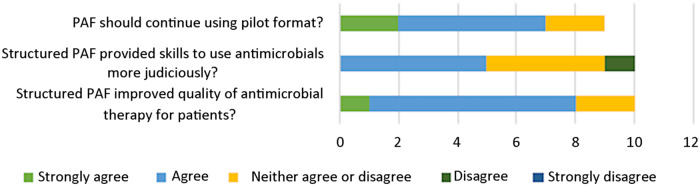
Responses to 5-point Likert scale questions.

The overall response rate was 25% (n = 10). With respect to demographics (Table 3), 60% of respondents were staff surgeons, and the rest were residents. Slightly more respondents had 7 or more years of practice (40%) compared to those with less years of practice (1–2 years and 3–4 years). All respondents attended 1–6 PAF session(s). Half of the respondents agreed that structured PAF provided them with skills to use antimicrobials more judiciously, and 80% of respondents agreed that structured PAF improved the quality of antimicrobial treatment for their patients (Fig. 3). Overall, 70% of respondents agreed that this pilot program should continue, and no respondent was against it. In terms of open-ended questions (Table 4), the most useful aspects of structured PAF served as a reminder to reassess duration and as a scheduled review of patients on antimicrobials. In contrast, respondents found the least useful aspects of face-to-face PAF to be the lack of a more formal structure and inconvenience of in-person rounds. In terms of barriers, respondents found it difficult to use antimicrobials judiciously due to lack of high-quality evidence, unique cases with lack of source control, and a high number of prescribers with differing prescribing habits. Conversely, improved dissemination of evidence-based guidelines based on local resistance rates was thought to facilitate judicious antimicrobial prescribing.

**Table 3.** Responses to Demographic Questions

Demographic Question	No. of Responses (n=10)
**Position on ACS team**	
Staff surgeon	6
Senior resident	2
Junior resident	2
**Years of practice/training**	
≥7 y	4
3–4 y	3
1–2 y	3
**No. of PAF attended**	
1–3 sessions	5
4–6 sessions	5

Note. PAF, Prospective audit and feedback; ACS, acute-care surgery.

**Table 4.** Responses to Open-Ended Questions

Question	Themes
Most useful aspect of PAF	Scheduled review of patients on antimicrobials
	Reminder to reassess duration
Least useful aspect of PAF	Lack of set time on Mondays for PAF
	Lack of portability of recommendations (ie, prefer email format)
Barriers to using antimicrobials more judiciously	Unique patient cases
	Lack of source control
	Questionable duration despite source control
	Lack of high-quality evidence
	Expanded number of prescribers
Facilitators to using antimicrobials more judiciously	Guidelines based on evidence and local resistance patterns
	Improved dissemination of guidelines

Note. PAF, prospective audit and feedback.

## Discussion

To our knowledge, this is the first study to compare 2 models of PAF in the surgical population. The quantitative aspect of our study detected no significant changes in level or trend in antimicrobial usage for all antimicrobials and targeted antimicrobials when comparing face-to-face and ad hoc PAF. Additionally, there were no significant differences in any of the secondary outcomes. Previous studies comparing PAF to no PAF found reductions in antimicrobial usage and length of stay.^9–11^ Taking this into account, our findings suggest that the format of PAF in surgical populations seems not to be as important as having some form of PAF. However, an important limitation of our study was the lack of a true preintervention period. Structured face-to-face PAF was pilot tested shortly after the establishment of the ACS service given the available ASP resources at the time. Therefore, no data representing antibiotic prescribing practices prior to PAF were available for comparison. Thus, sustained educational knowledge and judicious antimicrobial prescribing likely carried over to the ad hoc PAF period, which likely resulted in no difference in antimicrobial usage in both structured and ad hoc PAF periods. In addition, our study included only the surgical patients on the ACS team, who tend to have a short, uncomplicated admission compared to other surgical patients on non-ACS teams. Therefore, our study did not show a difference in length of stay with either format of PAF, compared to previous studies that included all surgical patients and compared PAF to no PAF.

Our qualitative survey found good support among respondents to continue structured face-to-face PAF because it facilitated knowledge and skills for judicious antimicrobial prescribing and optimized antimicrobial treatment for ACS patients. Given that 80% of respondents found that scheduled PAF improved antimicrobial usage but only 50% of respondents felt that they learned how to use antimicrobials more appropriately, there could be opportunities to further improve the educational goals of PAF among these prescribers. Additionally, it served as a reminder for the ACS team to reassess duration of antimicrobials for their patients. However, some respondents found the face-to-face structure inconvenient, and others preferred a more formal structure (ie, same time for PAF every Monday). Some barriers to judicious antimicrobial prescribing were similar to those reported in previous studies,^2,3^ such as unique cases and difficulty in determining source control. Additionally, one barrier that was highlighted in our survey was the high number of prescribers (ie, turnover of surgical residents). These surgical residents may have varied prescribing practices and less exposure to PAF and its educational benefits given their relatively short time on the ACS service. Conversely, improved dissemination of evidence-based guidelines was thought to facilitate judicious antimicrobial prescribing. At our institution, guidelines on the management of surgical-site infections are readily available on our intranet homepage through a unique ASP icon. Another limitation of our study was the small number of survey respondents; therefore, perceptions of our face-to-face PAF pilot program could be further explored.

Structured face-to-face PAF showed similar clinical outcomes, including no change in antimicrobial usage, to ad hoc PAF for ACS patients at our institution. In addition, structured PAF appeared to be well received and perceived as beneficial by surgical staff. Other educational antimicrobial stewardship interventions in addition to ad hoc PAF can be explored to better support judicious antimicrobial use in emergency general surgery.

## References

[1] 2021 Antibacterial agents in clinical and preclinical development: an overview and analysis. World Health Organization website. https://www.who.int/publications-detail-redirect/9789240047655/. Published 2022. Accessed May 16, 2023.

[2] Sartelli M , Duane TM , Catena F , Tessier JM , et al. Antimicrobial stewardship: a call to action for surgeons. Surg Infect 2016;17:625–631. 10.1089/sur.2016.187 PMC5124748 27828764

[3] Knox MC , Edye M. Educational antimicrobial stewardship intervention ineffective in changing surgical prophylactic antibiotic prescribing. Surg Infect 2016;17:224–228. 10.1089/sur.2015.194 26719984

[4] Sartelli M , Labricciosa FM , Scoccia L , et al. Nonrestrictive antimicrobial stewardship program in a general and emergency surgery unit. Surg Infect 2016;17:485–490. 10.1089/sur.2016.015 27135794

[5] Van Kasteren ME , Mannien J , Kullberg BJ , et al. Quality improvement of surgical prophylaxis in Dutch hospitals: evaluation of a multisite intervention by time series analysis. J Antimicrob Chemother 2005;56:1094–1102. 16234334 10.1093/jac/dki374

[6] Saied T , Hafez SF , Kandeel A , et al. Antimicrobial stewardship to optimize the use of antimicrobials for surgical prophylaxis in Egypt: a multicenter pilot intervention study. Am J Infect Control 2015;43:e67–e71. 26315059 10.1016/j.ajic.2015.07.004

[7] Duane TM , Zuo JX , Wolfe LG , et al. Surgeons do not listen: evaluation of compliance with antimicrobial stewardship program recommendations. Am Surg 2013;79:1269–1272. 24351354

[8] Langford BJ , Nisenbaum R , Brown KA , Chan A , et al. Antibiotics: easier to start than to stop? Predictors of antimicrobial stewardship recommendation acceptance. Clin Microbiol Infect 2020;26:1638–1643. 32771646 10.1016/j.cmi.2020.07.048

[9] Cakmacki M. Antimicrobial stewardship programmes and the surgeon’s roles. J Hosp Infect 2015;89:264–266. 25744279 10.1016/j.jhin.2015.01.006

[10] Davey P , Marwich CA , Scott CL et al. Interventions to improve antibiotic prescribing practices for hospital inpatients. Cochrane Database Syst Rev 2017;2:CD003543. 28178770 10.1002/14651858.CD003543.pub4 PMC6464541

[11] Guerri-Fernandez R , Villar-Garcia J , Herrera-Fernandez S , et al. An antimicrobial stewardship program reduces antimicrobial therapy duration and hospital stay in surgical wards. Rev Esp Quimioter 2016;29:119–122. 27167764

[12] Manuel-Vazquez A , Palacios-Ortega F , Garcia-Septiem J , et al. Antimicrobial stewardship programs are required in a department of surgery: “how” is the question a quasi-experimental study: results after three years. Surg Infect 2020;21:35–42. 10.1089/sur.2018.311 31347989

[13] Bernal JL , Cummins S , Gasparrini A. Interrupted time-series regression for the evaluation of public health interventions: a tutorial. Int J Epidemiol 2017;46:348–355. Erratum in: Int J Epidemiol 2020;49:1414. 27283160 10.1093/ije/dyw098 PMC5407170

[14] Shardell M , Harris AD , El-Kamary SS , Furuno JP , Miller RR , Perencevich EN. Statistical analysis and application of quasi experiments to antimicrobial resistance intervention studies. Clin Infect Dis 2007;45:901–907. 17806059 10.1086/521255

[15] Huitema BE McKean JW . Design specifications issues in time-series intervention models. Educ Psych Measure 2000;60:38–58.

